# Expression of the Renin-Angiotensin System in the Heart, Aorta, and Perivascular Adipose Tissue in an Animal Model of Type 1 Diabetes

**DOI:** 10.3390/ijms26199538

**Published:** 2025-09-29

**Authors:** Beatriz Martín-Carro, Sara Fernández-Villabrille, Paula Calvó-García, Nerea González-García, Francisco Baena-Huerta, Angie Hospital-Sastre, Pedro Pujante, Francisco José López-Hernández, Manuel Naves-Díaz, Sara Panizo, Natalia Carrillo-López, Cristina Alonso-Montes, José Luis Fernández-Martín

**Affiliations:** 1 Metabolismo Óseo, Vascular y Enfermedades Inflamatorias Crónicas, Instituto de Investigación Sanitaria del Principado de Asturias (ISPA), Unidad de Metabolismo Óseo, Unidad de Gestión Clínica de Medicina Interna, Hospital Universitario Central de Asturias (HUCA), 33011 Oviedo, Spain; bmartincar.ibsal@saludcastillayleon.es (B.M.-C.); sarafv0012@gmail.com (S.F.-V.); pauletaa.95@hotmail.com (P.C.-G.); nereagonzalezgarcia04@gmail.com (N.G.-G.); fba99.06@gmail.com (F.B.-H.); angie.hospital@ispasturias.es (A.H.-S.); mnaves.huca@gmail.com (M.N.-D.); cristina.alonso@ispasturias.es (C.A.-M.); jlfernandez.huca@gmail.com (J.L.F.-M.); 2 Department of Cardiology, University Hospital of Salamanca, Instituto de Investigación Biomédica de Salamanca (IBSAL), 37007 Salamanca, Spain; 3 Centro de Investigación Biomédica en Red de Enfermedades Cardiovasculares (CIBER-CV), Instituto de Salud Carlos III, 28029 Madrid, Spain; 4 Redes de Investigación Cooperativa Orientadas a Resultados en Salud (RICORS), RICORS2040-Renal, 28029 Madrid, Spain; 5 Endocrinología, Nutrición, Diabetes y Obesidad (ENDO), Instituto de Investigación Sanitaria del Principado de Asturias (ISPA), Servicio de Endocrinología y Nutrición, Hospital Universitario Central de Asturias (HUCA), 33011 Oviedo, Spain; pedropujanteal@gmail.com; 6 Translational Research on Renal and Cardiovascular Diseases (TRECARD), Instituto de Investigación Biomédica de Salamanca (IBSAL), Department of Physiology and Pharmacology, Universidad de Salamanca, 37007 Salamanca, Spain; flopezher@usal.es

**Keywords:** renin-angiotensin system, inflammation, heart, aorta, PVAT, diabetes, T1D

## Abstract

This study examined the expression of the renin-angiotensin system (RAS) and inflammatory markers in cardiovascular complications associated with long-term type 1 diabetes (T1D) using a rat model. After 24 weeks of streptozotocin-induced T1D, the animals exhibited metabolic alterations indicative of both cardiac and renal dysfunction. Tissue-specific dysregulation of RAS components and pro-inflammatory markers were observed in the heart, aorta, and perivascular adipose tissue (PVAT). In the heart, there was a significant upregulation of both classical (AT1R, 1.00 (0.22) vs. 1.70 (0.45) R.U.) and counter-regulatory RAS components (ACE2, 1.00 (0.43) vs. 1.96 (0.67) R.U.; *p* < 0.001) and MasR (1.00 (0.56) vs. 1.33 (0.29) R.U.; *p* = 0.004). The aorta displayed increased expression of classical RAS components alongside a significant reduction in ACE2 expression (1.00 (0.74) vs. 0.51 (0.48) R.U.; *p* < 0.032). Notably, PVAT showed a significant overexpression of classical RAS components (ACE 1.00 (0.22) vs. 4.08 (1.32) R.U.; *p* < 0.001, AT1R 1.00 (0.59) vs. 7.22 (4.14) R.U.; *p* < 0.001) and MasR (1.00 (0.70) vs. 4.52 (1.91) R.U.; *p* < 0.001), accompanied by increased expression of TNFα and ADAM17. These findings suggest that long-term T1D induces tissue-specific activation patterns of the RAS and inflammatory pathways within the cardiovascular system, which may contribute to the progression of diabetic cardiovascular complications. Therapeutic targeting of RAS components may represent a viable strategy for mitigating cardiovascular damage in T1D.

## 1. Introduction

Type 1 diabetes (T1D) represents a major global health burden, primarily due to its association with severe micro- and macrovascular complications [1]. Among these, cardiovascular disease constitutes the leading cause of morbidity and mortality in diabetic patients [1,2,3]. Emerging evidence indicates that inflammation plays a pivotal role in the pathogenesis and progression of these vascular complications [4,5,6,7]

The renin-angiotensin system (RAS), a critical regulator of blood pressure and fluid homeostasis, has emerged as a central contributor to diabetic inflammation [8,9,10] and cardiovascular dysfunction [11,12]. The classical RAS axis, involving renin, angiotensin-converting enzyme (ACE), and angiotensin II (Ang II) acting through the Ang II type 1 receptor (AT1R), promotes vasoconstriction, aldosterone secretion, and pro-inflammatory responses, thereby facilitating cardiovascular remodelling [13,14]. In contrast, a counter-regulatory arm of the RAS, which includes ACE2, Ang-(1–9), Ang-(1–7), and signaling through the Mas receptor (MasR) or Ang II type 2 receptor (AT2R), opposes these effects by promoting vasodilation and reducing inflammatory actions [8,15].

RAS components are expressed not only systemically but also locally within tissues, including the heart, blood vessels, and perivascular adipose tissue (PVAT), where they exert paracrine and autocrine effects while interacting with the systemic RAS [16,17,18]. Local Ang II production in the heart and aorta contributes to cardiac dysfunction, hypertension, and atherosclerosis [18,19,20]. PVAT, which surrounds vascular adventitia, influences vascular function and expresses multiple RAS components [21,22,23,24,25,26]. In obesity and metabolic disorders, PVAT-derived Ang II exacerbates inflammation and endothelial dysfunction, thereby increasing cardiovascular risk.

The RAS plays a pivotal role in cardiovascular complications associated with both T1D and type 2 diabetes (T2D) [27]. Differences in their pathophysiological mechanisms—insulin resistance in T2D and insulin deficiency in T1D [1]—may lead to distinct patterns of RAS component expression within tissues such as the heart, aorta, and PVAT. Although obesity is not a predominant factor in T1D, diabetes-induced metabolic dysregulation can nonetheless initiate PVAT inflammation, potentially altering the secretion of inflammatory adipokines that interact with the RAS to exacerbate vascular inflammation [28].

Despite the recognized significance, research on RAS alterations in T1D remains limited, with most studies focusing on T2D. Comparative analyses of the heart, aorta, and PVAT across both diabetes types are currently lacking, resulting in an incomplete understanding of tissue-specific interactions. Notably, to our knowledge, no studies have simultaneously assessed RAS alterations in all three tissues.

Understanding tissue-specific RAS expression, particularly within the heart, aorta, and PVAT, is crucial for elucidating their roles in cardiovascular physiology and pathology [17,29,30]. However, the complex interplay between local and systemic RAS complicates the delineation of their respective contributions [31]. The present study aims to investigate the expression of RAS in mediating inflammation within the heart, aorta, and PVAT in a long-term rat model of T1D.

## 2. Results

After 24 weeks of diabetes induction, rats exhibited significantly lower body weight (541 (76) g vs. 382 (22) g; *p* < 0.001) and lower relative weight of the perivascular adipose tissue (57 (15) mg/100 g BW vs. 21 (9) mg/100 g BW; *p* < 0.001) compared to control rats.

Diabetic rats showed significantly higher levels of serum and urinary glucose, HbA1c, and urine volume compared to control rats. Diabetic rats also exhibited significantly higher levels of serum creatinine and urea, along with urinary protein and urinary albumin-creatinine ratio (uACR), with no significant changes in creatinine clearance.

The results regarding lipid profile markers showed that in diabetic rats, triglyceride (TG) levels were higher compared to control rats, while no significant differences were observed in total cholesterol and high-density lipoprotein (HDL-C) levels between the groups.

Plasma levels of renin-angiotensin components: ACE, ACE2, and AngII, and N-terminal pro-B-type natriuretic peptide (NT-proBNP) were higher in diabetic rats compared to the control group (Table 1).

The gene expression of classical (*Ace*, *At1r*) and counter-regulatory (*Ace2*, *At2r*, MasR) components of the renin-angiotensin system, as well as inflammatory markers as tumor necrosis factor alpha (*Tnfα*) and a disintegrin and metalloprotease 17 (*Adam17*), also known as *Tace* (tumor necrosis factor-α-converting enzyme), in the heart, aorta, and PVAT is detailed in Table 2.

**Figure 1:**
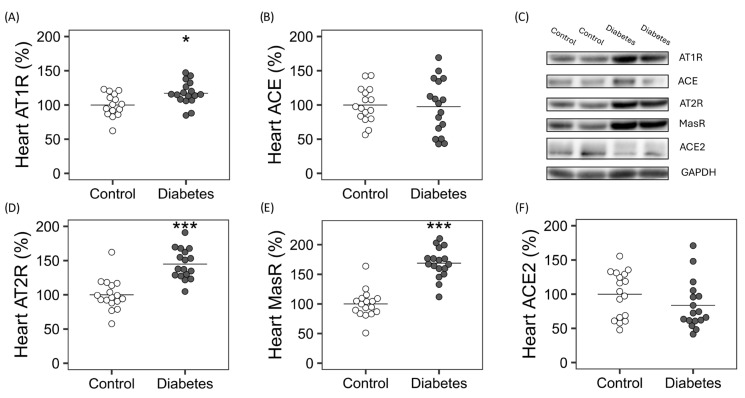
Heart protein expression of markers of the classical and counter-regulatory arm of the renin-angiotensin system. Relative quantification of (**A**) AT1R: angiotensin II type 1 receptor, (**B**) ACE: angiotensin-converting enzyme, (**D**) AT2R: angiotensin II type 2 receptor, (**E**) MASR: Mas receptor, and (**F**) ACE2: angiotensin-converting enzyme 2, and (**C**) representative image of Western Blot analysis. Non-parametric Wilcoxon rank sum test was used. Horizontal line represents mean. * *p* ≤ 0.05 Vs. Control, *** *p* ≤ 0.001 Vs. Control.

In the heart, gene and/or protein expression of classical RAS markers (Table 2 Part A and Figure 1A,B, respectively), such as renin mRNA (1.00 (0.68) vs. 1.95 (1.41) R.U.; *p* < 0.05;) and *At1r* mRNA and protein (1.00 (0.22) vs. 1.70 (0.45) R.U.; *p* < 0.001; 100.00 (17.00) vs. 117.20 (16.86) %; *p* < 0.05) (Table 2 Part A and Figure 1A), were significantly higher in diabetic rats. In contrast, no differences in *Ace* mRNA or protein levels were found between the groups (Table 2 Part A and Figure 1B).

Regarding the expression of markers of counter-regulatory arm of RAS (Figure 1D,F), the heart of diabetic rats showed higher protein levels of AT2R (100.00 (22.68) vs. 144.95 (22.25) %; *p* < 0.001) (Figure 1D) and MASR (100.00 (23.56) vs. 168.83 (25.44) %; *p* < 0.001) (Figure 1E), with no changes in ACE2 protein levels (Figure 1F) but higher mRNA levels (1.00 (0.43) vs. 1.96 (0.67) R.U.; *p* < 0.001) (Table 2 Part A).

**Figure 2:**
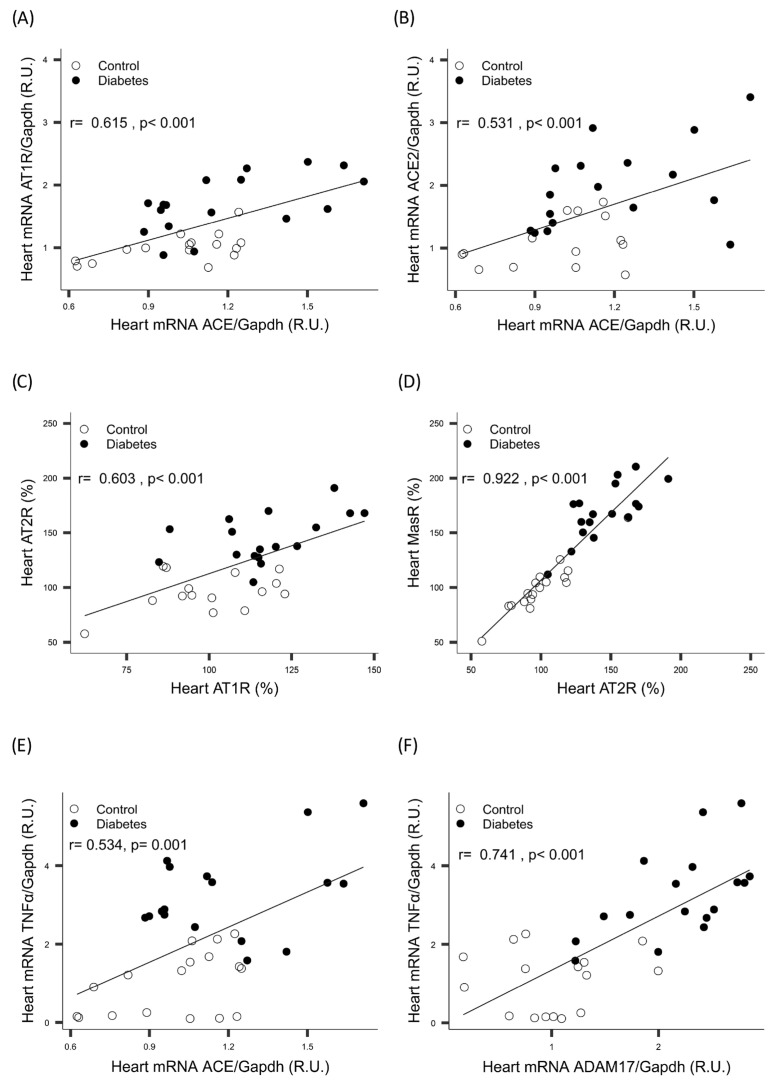
Heart correlation between *Ace* mRNA and both (**A**) *At1r*: angiotensin II type 1 receptor mRNA and (**B**) *Ace2*: angiotensin-converting enzyme 2 mRNA, between *At2r*: angiotensin II type 2 receptor protein and both (**C**) *At1r*: angiotensin II type 1 receptor protein and (**D**) MASR: Mas receptor protein, and between *Tnfα*: tumor necrosis factor alpha mRNA and both (**E**) *Ace2* mRNA and (**F**) *Adam17*: a disintegrin and metalloprotease 17 mRNA. *Gapdh*: glyceraldehyde-3-phosphate dehydrogenase.

Linear regression analysis showed a positive correlation between *Ace* mRNA and both *At1r* and *Ace2* mRNA. Additionally, a positive correlation was found between protein levels of AT2R and both AT1R and MASR protein levels, which remained significant even after adjusting for diabetes (Figure 2A–D).

In the aorta (Table 2 Part B), gene expression of the classical RAS markers, *Ace* (1.00 (0.62) vs. 1.90 (1.03) R.U.; *p* < 0.05) and *At1r* (1.00 (0.59) vs. 1.74 (0.91) R.U.; *p* < 0.05), was higher in diabetic rats, while the counter-regulatory RAS marker, *Ace2* (1.00 (0.74) vs. 0.51 (0.48) R.U.; *p* < 0.05), was lower, compared to controls; no differences in *Atr2* or *MasR* mRNA levels were found between the groups.

**Figure 3:**
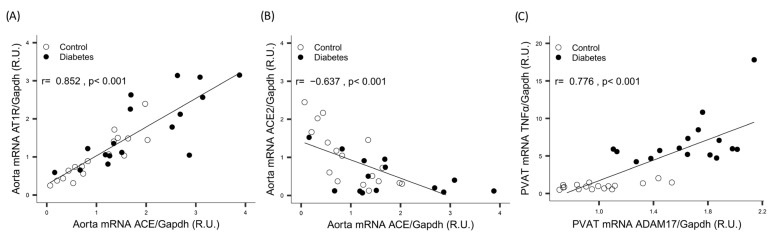
Aorta correlation between *Ace*: angiotensin-converting enzyme mRNA and both (**A**) *At1r*: angiotensin II type 1 receptor mRNA and (**B**) *Ace2*: angiotensin-converting enzyme 2 mRNA. (**C**) PVAT correlation between *Adam17*: a disintegrin and metalloprotease 17 mRNA and Tnfα mRNA. *Gapdh*: glyceraldehyde-3-phosphate dehydrogenase.

A significant positive correlation was observed between *Ace* and *At1r*, while a negative correlation was found between *Ace* and *Ace2* (Figure 3A,B).

In the PVAT (Table 2 Part C), gene expression of *Ace* (1.00 (0.22) vs. 4.08 (1.32) R.U.; *p* < 0.001), *At1r* (1.00 (0.59) vs. 7.22 (4.14) R.U.; *p* < 0.001) and *MasR* (1.00 (0.70) vs. 4.52 (1.91) R.U.; *p* < 0.001) was higher in diabetic rats, with no change in either *Ace2* or *At2r* between the two groups.

### Inflammation Markers

Gene expression of *Tnfα* and *Adam17* was significantly higher in the heart (Table 2 Part A) and PVAT (Table 2 Part C) of diabetic rats compared to control rats. In the aorta (Table 2 Part B), although there were no differences in *Tnfα* gene expression between the two groups, *Adam17* mRNA levels were higher in the diabetic rats (1.00 (0.43) vs. 1.47 (0.61) R.U.; *p* < 0.05).

In the heart, a significant positive correlation was found between *Tnfα* and both *Ace* and *Adam17* (Figure 2E,F). In the PVAT, a significant positive correlation was found between *Adam17* and *Tnfα* (Figure 3C). All correlations persisted after adjustment for diabetes.

## 3. Discussion

This study showed that T1D may be associated with significant alterations in the RAS and inflammation within cardiovascular tissues, including the heart, aorta, and PVAT. These findings suggest specific patterns of RAS activation in the tissues that may contribute, at least in part, to cardiovascular dysfunction in diabetes. To our knowledge, this study is the first to analyse alterations in RAS components in the heart, aorta, and PVAT in a T1D experimental model, providing new insights into how these tissues interact and contribute to the cardiovascular complications associated with T1D.

The increase in plasma levels of ACE, ACE2, and Ang II observed in diabetic animals compared with controls may indicate activation of the RAS, as proposed by other authors [32,33], and is consistent with the findings in the tissues analyzed in this study. In the heart, diabetic rats exhibited upregulation of both classical (AT1R) and counter-regulatory (ACE2, AT2R, and MasR) RAS components. The higher AT1R expression suggests a possible heightened activation of the pro-inflammatory and pro-fibrotic axis of the RAS, in line with previous studies showing that Ang II, through its binding to AT1R, could promote cardiac hypertrophy, inflammation, and oxidative stress [34,35,36]. Given the well-established role of the RAS in the initiation and progression of inflammation, our results further support this connection.

In fact, the hearts of diabetic rats exhibited higher expression of *Tnfα* and *Adam17*. These findings are consistent with previous studies showing that angiotensin II, through binding to the AT1R receptor, enhances ADAM17 expression and activation, thereby promoting inflammation and hypertrophy [37,38]. Additionally, ADAM17 plays a critical role in modulating the local balance of angiotensin peptides by facilitating the release of the soluble form of ACE2 [39]. This is consistent with our results in the plasma of diabetic rats. Although the functional role of soluble ACE2 remains unclear, its increase has been linked to a higher risk of cardiovascular events [40,41]. The shedding of ACE2 could alter the balance between ACE and ACE2 activities, which may affect tissue and organ pathogenesis [39].

The increase in the expression of counter-regulatory components (ACE2, AT2R, and MasR) may represent a compensatory response to mitigate the harmful effects of AT1R activation. These findings align with evidence suggesting that the ACE2/Ang-(1–7)/MasR axis could have cardioprotective effects by reducing inflammation and oxidative stress [15,42,43,44]. Further supporting this notion, experimental studies in diabetic murine models have shown an upregulation of ACE2 accompanied by a downregulation of ACE in the heart, indicating an early protective shift that limits Ang II accumulation and enhances Ang-(1–7) formation, which may help delay cardiac remodelling and dysfunction [45]. However, it is important to note that this compensatory activation may predominate mainly during the early stages of diabetes. In more advanced phases, such as in our long-duration diabetic model, this regulation might be diminished or overridden by persistent activation of the classical RAS, shifting the balance toward pathological remodelling and cardiovascular deterioration observed in other studies [40,41] suggesting a dysregulated response. In addition, the compensatory activation may not have fully counteracted the pathological effects of AT1R, as suggested by the higher expression of *Tnfα* and *Adam17*. This is further supported by the presence of cardiomyocyte hypertrophy and increased fibrosis (evidenced by greater collagen deposition, elevated fibronectin and TGF-β1 levels, and activation of the pro-fibrotic Wnt/β-catenin pathway), which were also been observed in the same diabetic rats [46]. All these factors could have contributed to cardiac dysfunction, as reflected by increased NT-proBNP and ACE2 levels, both well-established biomarkers of myocardial dysfunction and cardiovascular events [40,41,47,48].

In the aorta, a clear predominance of the classical RAS pathway was observed, with significant upregulation of *Ace* and *At1r*, along with downregulation of *Ace2*. This imbalance may have promoted vascular inflammation, oxidative stress, and remodelling, key processes in the pathogenesis of atherosclerosis and hypertension in diabetes [49,50]. The negative correlation between *Ace* and *Ace2* further supports the notion of a disrupted equilibrium between the classical and counter-regulatory arms of the RAS in vascular tissues [12]. These findings suggest that the aorta could have been particularly susceptible to the pro-inflammatory effects of Ang II, which could have led to endothelial dysfunction and vascular stiffness [51].

PVAT showed significant overexpression of *Ace* and *At1r*, as well as *MasR*, accompanied by increased inflammatory markers *Tnfα* and *Adam17*. PVAT, an important regulator of vascular function, is known to produce RAS components in both physiological and pathological conditions. In diabetes, overactivation of PVAT-derived RAS could have exacerbated vascular inflammation and endothelial dysfunction, contributing to increased cardiovascular risk [52]. The upregulation of *Tnfα* and *Adam17* further emphasizes the inflammatory environment in PVAT, which could have propagated vascular damage through paracrine signalling. These findings are consistent with previous studies linking PVAT inflammation to metabolic disorders and cardiovascular complications [25,53]. The simultaneous upregulation of *MasR* suggests activation of the counter-regulatory RAS axis, which is typically associated with anti-inflammatory and vasoprotective effects. However, the coexistence of increased *At1r* and inflammatory markers suggests that *MasR* overexpression likely reflects a protective but insufficient compensatory response unable to counteract the pathological activation of the classical RAS. This imbalance may contribute to the persistence of vascular inflammation and dysfunction in chronic diabetes. Moreover, the greater ratio of expression changes observed in the analyzed RAS components supports the notion that PVAT dysfunction may be a key factor in vascular disease, as suggested by previous studies [54].

Taken together, the results suggest that T1D may induce tissue-specific dysregulation of the RAS, with predominant activation of the classical arm in the aorta and PVAT, and a more complex pattern in the heart, where counter-regulatory components were also upregulated.

These findings highlight the need for tissue-specific therapeutic strategies targeting RAS components to mitigate cardiovascular complications in T1D.

While our findings show a simultaneous overexpression of pro-inflammatory markers alongside alterations in the expression of classical and counter-regulatory RAS components in the heart, aorta, and perivascular adipose tissue, it is important to acknowledge that this study is primarily descriptive. Therefore, it does not provide direct mechanistic evidence of the interaction between RAS activation and inflammation contributing to cardiovascular damage in this experimental model. Nevertheless, this co-expression suggests a potential interplay between the two pathways in diabetic cardiovascular tissues, a hypothesis supported by previous papers [9,10,55,56] and provides valuable information for future research. Future investigations should incorporate interventions targeting inhibition of the systemic renin–angiotensin system (RAS)—including ACE inhibitors, AT1 receptor antagonists, or Ang-(1–7) analogs—and/or selective inhibition of tissue-specific RAS activity in the heart, aorta, and perivascular adipose tissue (PVAT), to directly determine whether modulation of this pathway influences inflammatory processes and cardiovascular functional outcomes.

This study presents other limitations that should be considered when interpreting the results.

First, no direct functional analyses -such as echocardiography or vascular reactivity tests-were performed, which would have allowed for a more comprehensive assessment of cardiovascular involvement and strengthened the interpretation of the molecular and histological findings.

Second, this study did not include experimental groups receiving RAS blocker treatment, which limits the possibility of assessing how such interventions might influence inflammation and cardiovascular function in this model. This limitation underscores the importance of future studies to functionally confirm the role of the RAS in the development of cardiovascular complications in diabetes

Finally, a general limitation of preclinical diabetes studies is that no animal model fully replicates the complexity and progressive nature of the human disease. Although our long-term T1D model allows the exploration of chronic cardiovascular alterations under clinically relevant conditions, future comparative studies with other models, including those of type 2 diabetes, will be essential to better define the specific role of the renin-angiotensin system depending on the type and progression of the disease.

Overall, while the study provides relevant insights into tissue-specific RAS dysregulation in type 1 diabetes, further investigations are needed to mechanistically explore the links between RAS activation, inflammation, and cardiovascular damage, as well as the therapeutic potential of RAS modulators in this context.

## 4. Materials and Methods

### 4.1. Animals

Four-month-old male Wistar rats weighing 425 ± 43 g (n = 34) were housed in a climate-controlled room with a 12 h light/dark cycle and were fed ad libitum with standard rodent chow and water. Animal handling was performed according to European Union laws and experimental procedures were approved by the Research Ethics Committee of the Oviedo University (PROAE 26/2021).

### 4.2. Experimental Design

The experimental design has been previously detailed [46]. Briefly, type 1 diabetes (T1D) was induced in fasted rats (n = 22) by administering a single intraperitoneal injection of streptozotocin (55 mg/Kg) dissolved in 0.1 M citrate buffer. Control rats (n = 17) received an equal volume of citrate buffer. The injections were performed under anaesthesia with 2% isoflurane. After one week, the rats with glycaemia higher than 350 mg/dL for three consecutive days were considered diabetic and included in the study. Glycaemia and body weight (BW) were measured throughout the study. Long-acting biosynthetic human insulin (Lantus^®^, Aventis Pharma, Bad Soden, Germany) (1–2 IU) and/or insulin pellets (0.5 IU/24 h slow-release) (Linshin, Toronto, ON, Canada) were administered when blood glucose levels exceeded 500 mg/dL, ketone bodies were over 3 mmol/L, and/or there was significant body weight loss (>10% from baseline). Insulin pellets were administered when long-acting insulin failed to reverse weight loss. In the final analysis, 17 diabetic rats were included (one animal was excluded due to glucose levels were below 350 mg/dL, and four died during the study).

The total duration of the experiment was 24 weeks. One day prior to sacrifice, rats were placed in metabolic cages for 24 h urine collection. The urine volume was measured, centrifuged at 2500 rpm for 5 min, and the clear supernatant was stored at −80 °C. The rats were sacrificed by exsanguination under isoflurane anaesthesia. Immediately after sacrifice, blood was collected in tubes with or without EDTA, centrifuged at 3000 rpm for 15 min at 4 °C to obtain plasma or serum, and stored at −80 °C until further analysis. The heart, aorta and periaortic adipose tissue (PVAT) were immediately removed, weighed, and stored at −80 °C until analysis.

### 4.3. Serum/Plasma and Urine Measurements

Serum levels of glucose, proteins, albumin, creatinine, total cholesterol, triglycerides (TG), high-density lipoprotein cholesterol (HDL-C), and urea were measured using a multichannel autoanalyzer (Hitachi 717; Boehringer Mannheim, Berlin, Germany). Plasma levels of ACE (ER0094, Wuhan Fine Biotech, Co., Ltd., Wuhan, China), ACE2 (ER0609, Wuhan Fine Biotech, Co., Ltd.), Ang II (ER1637, Wuhan Fine Biotech, Co., Ltd.) and NT-proBNP (CSB-E08752r, Cusabio, Houston, TX, USA) were assessed using a commercial enzyme-linked immunosorbent assay (ELISA) kits following the manufacturer’s recommendations. Blood Hb1Ac was measured at sacrifice using the A1Cnow+^®^ device (PTS Diagnostics, Indianapolis, IN, USA).

Urinary biomarkers were expressed as a ratio to urinary creatinine. Creatinine clearance and uACR were calculated using the following formulas: creatinine clearance (mL/min) = urinary creatinine (mg/dL) × urinary volume (mL)/serum creatinine (mg/dL) × 1440 (min); uACR (mg/g) = urinary albumin (mg/L)/urinary creatinine (g/L).

### 4.4. RNA Extraction and Quantitative Real-Time PCR

Total RNA was extracted from the heart, aorta, and PVAT using TRI-Reagent (Sigma-Aldrich, St. Louis, MO, USA). cDNA was synthesized from 1 µg of total RNA using a high-capacity cDNA reverse transcription kit (Applied Biosystems, Waltham, MA, USA). Quantitative real-time PCR was performed to measure mRNA levels of Ace (Rn00561094, Thermo Fisher Scientific, Waltham, MA, USA), Ace2 (Rn01416293, Thermo Fisher Scientific), At1r (503930, Roche, Basel, Switzerland), At2r (Rn00560677, Thermo Fisher Scientific), MasR (Rn00562673, Thermo Fisher Scientific), Renin (Rn02586313, Thermo Fisher Scientific), Tnfα (Rn99999017, Thermo Fisher Scientific), and Adam17 or Tace (Rn00571880, Thermo Fisher Scientific), using TaqMan Universal PCR Master Mix (Thermo Fisher Scientific) in a QuantStudio 3 Real-Time PCR System (Applied Biosystems). Glyceraldehyde-3-phosphate dehydrogenase (Gapdh) (Rn99999916, Thermo Fisher Scientific) was used for normalization. The ∆∆CT method was used to quantify the relative expression of each gene [57].

### 4.5. Immunoblotting

Heart tissue was homogenized in RIPA buffer. After centrifugation at 14,000 rpm for 20 min at 4 °C, the supernatant was collected, and total protein content was measured by the DC protein assay reagents (Bio-Rad, Hercules, CA, USA). For each assay, 40 μg of protein was separated by sodium dodecyl sulphate–polyacrylamide gel (10%) electrophoresis (SDS-PAGE) and transferred onto polyvinylidene difluoride (PVDF) membranes (Amersham Hybond, Amersham Biosciences, Amersham, UK). Transfer efficiency was checked by Ponceau red staining (Sigma-Aldrich). The membranes were incubated with specific antibodies according to the manufacturer’s instructions: ACE (dilution 1:1000; #ab254222, Abcam, Cambridge, UK); ACE2 (dilution 1:1000; #ab108252, Abcam); AT1R (dilution 1:1000; #PB9470, Boster Bio, Wuhan, China); AT2R (dilution 1:1000; #ab92445, Abcam); MasR (dilution 1:500; #ab200685, Abcam).

After washing, membranes were incubated with peroxidase-conjugated secondary antibodies (Santa Cruz, Dallas, TX, USA), detected using the ECL Western blotting Detection Kit (Bio-Rad) and the ChemiDoc Gel Imaging System Model XRS (Bio-Rad), and quantified using Quantity One 1-D Analysis Software Version 4 (Bio-Rad). All data were normalized to the housekeeping protein GAPDH (dilution 1:3000; sc-25778, Santa Cruz Biotechnology, Dallas, TX, USA) and expressed as a percentage of control values.

### 4.6. Statistical Analysis

The sample size was calculated based on an expected effect size of 1, a statistical power of 80%, and a type I error rate of 0.05 for all quantitative variables in the study. This calculation resulted in a required sample size of 17 animals per group. To account for potential mortality or failure to achieve blood glucose levels above 350 mg/dL, 5 additional animals were included in the diabetic rat group. Results were expressed as mean and standard deviation (SD). Differences between diabetic and control rats were tested using the non-parametric Wilcoxon rank sum test. Correlations between continuous variables were assessed using linear regression. Significant differences were considered when *p* < 0.05. The statistical program used was R version 4.4.0.

## 5. Conclusions

The changes observed in the heart, aorta, and PVAT are accompanied by an imbalance in local RAS. Given the known contribution of the RAS to the inception and development of inflammation, our results suggest that cardiovascular complications in diabetes could be driven by an imbalance in local RAS. These findings support the idea that targeting the RAS, along with anti-inflammatory therapies, could offer potential therapeutic strategies for preventing or mitigating diabetic complications, particularly those related to the heart. Future studies investigating the mechanisms linking RAS activation, inflammation, and tissue damage in diabetes are needed to further elucidate the pathophysiology of the disease and identify novel treatment approaches.

## Figures and Tables

**Table 1:** Biochemical parameters in control and diabetic rats.

	Control (n = 17)	Diabetes (n = 17)	*p*-Value
Serum Glucose (mg/dL)	193.04 (19.20)	693.32 (60.94)	<0.001
HbA1c (%)	4.87 (0.38)	14.00 (0.00)	<0.001
Serum Proteins (g/L)	61.92 (3.76)	55.58 (2.26)	<0.001
Serum Albumin (g/L)	41.99 (3.19)	34.89 (2.10)	<0.001
Serum Creatinine (mg/dL)	0.32 (0.04)	0.37 (0.06)	0.009
Serum Urea (mg/dL)	26.02 (4.29)	35.21 (6.63)	<0.001
Serum total cholesterol (mg/dL)	84.47 (16.59)	84.38 (14.71)	0.667
Serum HDL-C (mg/dL)	55.55 (13.71)	60.51 (6.50)	0.148
Serum TG (mg/dL)	126.36 (56.04)	199.50 (101.23)	0.021
Plasma ACE (ng/mL)	17.16 (3.24)	23.32 (4.14)	<0.001
Plasma ACE2 (ng/mL)	31.96 (7.98)	41.29 (9.23)	0.004
Plasma AngII (pg/mL)	542.29 (86.83)	695.68 (84.70)	<0.001
Plasma NT-proBNP (µg/mL)	251.30 (76.70)	304.78 (54.09)	0.034
Urine volume (mL)	10.34 (5.44)	150.53 (28.34)	<0.001
Urinary Glucose (mg/24 h)	20.79 (27.57)	15,216.63 (2633.44)	<0.001
Urinary proteins (mg/mg)	0.72 (0.18)	1.14 (0.40)	<0.001
uACR (mg/g)	11.42 (10.30)	39.09 (13.48)	<0.001
Creatinine clearance (mL/min/kg)	3.03 (0.64)	3.39 (0.99)	0.480

HbA1c: Glycosylated haemoglobin; HDL-C: high-density lipoprotein cholesterol; TG: triglycerides; ACE: Angiotensin-converting enzyme; ACE2: Angiotensin-converting enzyme 2; AngII: Angiotensin II; NT-proBNP: N-terminal pro-B-type natriuretic peptide; uACR: Urinary albumin/creatinine ratio. Non-parametric Wilcoxon rank sum test was used. Data are expressed as mean (SD).

**Table 2:** Gene expression of markers of the RAS and inflammation in (A) heart, (B) aorta, and (C) PVAT.

	(A) Heart			(B) Aorta			(C) PVAT		
	Control	Diabetes	*p*-Value	Control	Diabetes	*p*-Value	Control	Diabetes	*p*-Value
n	17	17		17	17		17	17	
*Ace* (R.U.)	1.00 (0.22)	1.19 (0.28)	0.113	1.00 (0.62)	1.90 (1.03)	0.014	1.00 (0.22)	4.08 (1.32)	<0.001
*Ace2* (R.U.)	1.00 (0.43)	1.96 (0.67)	<0.001	1.00 (0.74)	0.51 (0.48)	0.032	1.00 (0.65)	1.31 (0.81)	0.262
*At1r* (R.U.)	1.00 (0.22)	1.70 (0.45)	<0.001	1.00 (0.59)	1.74 (0.91)	0.017	1.00 (0.59)	7.22 (4.14)	<0.001
*At2r* (R.U.)	1.00 (1.72)	0.24 (0.29)	0.057	1.00 (0.95)	0.47 (0.24)	0.102	1.00 (1.84)	0.63 (0.51)	0.121
*MasR* (R.U.)	1.00 (0.56)	1.33 (0.29)	0.004	1.00 (0.57)	0.68 (0.20)	0.144	1.00 (0.70)	4.52 (1.91)	<0.001
*Tnfα* (R.U.)	1.00 (0.80)	3.25 (1.11)	<0.001	1.00 (0.50)	0.69 (0.47)	0.089	1.00 (0.41)	6.91 (3.34)	<0.001
*Adam17* (R.U.)	1.00 (0.51)	2.19 (0.53)	<0.001	1.00 (0.43)	1.47 (0.61)	0.029	1.00 (0.25)	1.65 (0.31)	<0.001

*Ace*: angiotensin-converting enzyme; *Ace2*: angiotensin-converting enzyme 2; *At1*/*2r*: angiotensin II type 1/2receptor; *MasR*: Mas receptor; *Tnfα*: tumor necrosis factor alpha; *Adam17*: a disintegrin and metalloprotease 17; *p*: *p*-value; PVAT: perivascular adipose tissue; R.U.: relative units. Non-parametric Wilcoxon rank sum test was used. Data are expressed as mean (SD).

## Data Availability

The original contributions presented in this study are included in the article. Further inquiries can be directed to the corresponding authors.

## Abbreviations

The following abbreviations are used in this manuscript:

ACE	Angiotensin-converting enzyme
ACE2	Angiotensin-converting enzyme 2
ADAM17	A disintegrin and metalloproteinase 17
Ang II	angiotensin II
AT1R	Angiotensin II type 1 receptor
AT2R	Angiotensin II type 2 receptor
GAPDH	Glyceraldehyde-3-phosphate dehydrogenase
Hb1Ac	Glycosylated haemoglobin
HDL-C	High-density lipoprotein cholesterol
MasR	Mas receptor
NT-proBNP	N-terminal pro-B-type natriuretic peptide
PVAT	Perivascular adipose tissue
RAS	Renin-angiotensin system
R.U.	Relative units
SD	Standard deviation
TG	Triglycerides
T1D	Type 1 diabetes
TNFα	Tumor necrosis factor-alpha
uACR	Albumin-to-creatinine ratio
